# Inactivation of *Bacillus anthracis* Spores during Laboratory-Scale Composting of Feedlot Cattle Manure

**DOI:** 10.3389/fmicb.2016.00806

**Published:** 2016-05-27

**Authors:** Shanwei Xu, Amanda Harvey, Ruth Barbieri, Tim Reuter, Kim Stanford, Kingsley K. Amoako, Leonard B. Selinger, Tim A. McAllister

**Affiliations:** ^1^Lethbridge Research and Develeopment Centre, Agriculture and Agri-Food Canada, LethbridgeAB, Canada; ^2^Department of Biological Sciences, University of Lethbridge, LethbridgeAB, Canada; ^3^Alberta Agriculture and Forestry, LethbridgeAB, Canada; ^4^Lethbridge Laboratory, Canadian Food Inspection Agency, National Centres for Animal Disease, LethbridgeAB, Canada

**Keywords:** *Bacillus anthracis*, spore, sporulation, compost, anthrax, temperature

## Abstract

Anthrax outbreaks in livestock have social, economic and health implications, altering farmer’s livelihoods, impacting trade and posing a zoonotic risk. Our study investigated the survival of *Bacillus thuringiensis* and *B. anthracis* spores sporulated at 15, 20, or 37°C, over 33 days of composting. Spores (∼7.5 log_10_ CFU g^-1^) were mixed with manure and composted in laboratory scale composters. After 15 days, the compost was mixed and returned to the composter for a second cycle. Temperatures peaked at 71°C on day 2 and remained ≥55°C for an average of 7 days in the first cycle, but did not exceed 55°C in the second. For *B. thuringiensis*, spores generated at 15 and 21°C exhibited reduced (*P* < 0.05) viability of 2.7 and 2.6 log_10_ CFU g^-1^ respectively, as compared to a 0.6 log_10_ CFU g^-1^ reduction for those generated at 37°C. For *B. anthracis*, sporulation temperature did not impact spore survival as there was a 2.5, 2.2, and 2.8 log_10_ CFU g^-1^ reduction after composting for spores generated at 15, 21, and 37°C, respectively. For both species, spore viability declined more rapidly (*P* < 0.05) in the first as compared to the second composting cycle. Our findings suggest that the duration of thermophilic exposure (≥55°C) is the main factor influencing survival of *B. anthracis* spores in compost. As sporulation temperature did not influence survival of *B. anthracis*, composting may lower the viability of spores associated with carcasses infected with *B. anthracis* over a range of sporulation temperatures.

## Introduction

Anthrax is a lethal mammalian disease, capable of infecting animals and humans and remains an ongoing global problem (Spencer, 2003; Sternbach, 2003). Before the development of a vaccine for the treatment of livestock in the 1870’s, anthrax was one of the leading causes of worldwide mortality in cattle, goats, horses, and sheep (Hugh-Jones and Blackburn, 2009). During the past 20 years, even with the availability of a vaccine, human anthrax is still a significant problem in Western Africa, Eastern Europe, and Central Asia, with sporadic outbreaks continuing to occur in livestock in the United States and Canada (Levin, 2014).

The causative agent of anthrax is *Bacillus anthracis*, a Gram-positive, aerobic, endospore forming rod-shaped bacterium (World Health Organization, 2008). Spores are the primary infectious form of *B. anthracis* with infection occurring through ingestion, inhalation or cutaneous exposure (Thappa and Karthikeyan, 2001). *Bacillus* spores are resistant to heat, desiccation, radiation and chemical treatment (Stephens, 1998) so decontamination can be a challenge. It has been reported that *Bacillus* spores can persist in soil for at least 300 years (Nicholson et al., 2000). The infectious dose of *B. anthracis* spores varies among host species and with route of infection. As few as ten inhaled *B. anthracis* spores are sufficient to cause infection in cattle and sheep (Smith, 1973), while 500–55,000 inhaled spores is the estimated range for a lethal dose for humans (Wallin et al., 2007).

In Canada, there is still a concern with repeated outbreaks of anthrax in wild bison populations in the Northwest Territories, Northern Alberta, Manitoba, and Saskatchewan (Beaumont, 2013; Elkin et al., 2013). Moreover, an anthrax outbreak occurred in Saskatchewan in 2006, resulting in the death of 804 livestock (Himsworth and Argue, 2008). Proper disposal of animal carcasses infected with *B. anthracis* is essential to minimize spore contamination and reduce the risk of transmission to animals and humans. In addition, the extreme virulence, environmental persistence and multiple routes of infection have also resulted in anthrax spores being employed as a biological weapon (Ala’Aldeen, 2001; Cole, 2010). Therefore, in the event of a natural or a terror related outbreak of *B. anthracis*, proper technologies are required to inactivate spores associated with contaminated livestock carcasses.

In Canada, current disposal practices approved by Canadian Food Inspection Agency (CFIA) for *B. anthracis* infected carcasses include incineration and deep burial with chemical treatment using 10% formalin or 5% sodium hydroxide (CFIA, 2013). However, Canada’s vast geographical area and transportation distances frequently make incineration impractical as a disposal method. Moreover, deep burial can render the disposal site a long-term reservoir of spores with heavy rain fall and soil saturation promoting renewed transmission as viable spores migrate to the soil surface (Nicholson, 2002; Himsworth and Argue, 2008). Composting may offer a practical and economical means for the safe disposal of carcasses during an anthrax outbreak. Composting is an aerobic decomposition process whereby organic matter is degraded by the actions of mesophilic and thermophilic bacteria and fungi. Compost is often alkaline (pH 8-10) as a result of the liberation of ammonia from the deamination of amino acids and temperatures can reach ∼70°C and exceed 55°C for weeks or even months (Xu et al., 2009; Stanford et al., 2015). These conditions have been shown to inactivate most microbial pathogens including *Listeria* (Erickson et al., 2009a), Shiga-toxigenic *Escherichia coli* (Xu et al., 2009), *Salmonella* (Erickson et al., 2009b), *Giardia, Cryptosporidium* (Van Herk et al., 2004), and avian influenza, Newcastle disease and foot-and-mouth disease viruses (Guan et al., 2009, 2010). Even recalcitrant proteins such as the prions associated with scrapie, chronic wasting disease (CWD) and bovine spongiform encephalopathy (BSE) are degraded during composting (Xu et al., 2014).

Our research group has previously used related spore-forming bacteria (i.e., *B. licheniformis, B. thuringiensis*, and *B. cereus*) as surrogates for investigating the inactivation of *B. anthracis* in compost under field conditions (Reuter et al., 2011; Stanford et al., 2015). To further define the feasibility of composting for disposal of *B. anthracis* infected carcasses, assessment of the fate of *B. anthracis* spores in compost is required. However, such studies with *B. anthracis* can only be safely conducted under full laboratory containment, conditions that we have met using specially designed laboratory-scale composters (Xu et al., 2010). Our recent findings have shown that sporulation temperature was a key factor influencing survival of *B. cereus* spores in cattle carcass compost (Stanford et al., 2015). Therefore, the objective of this study was to assess the survival of *B. anthracis* spores generated at different sporulation temperatures (15, 21, or 37°C) using laboratory composters in containment.

## Materials and Methods

### Laboratory Composting Experiment

**FIGURE 1:**
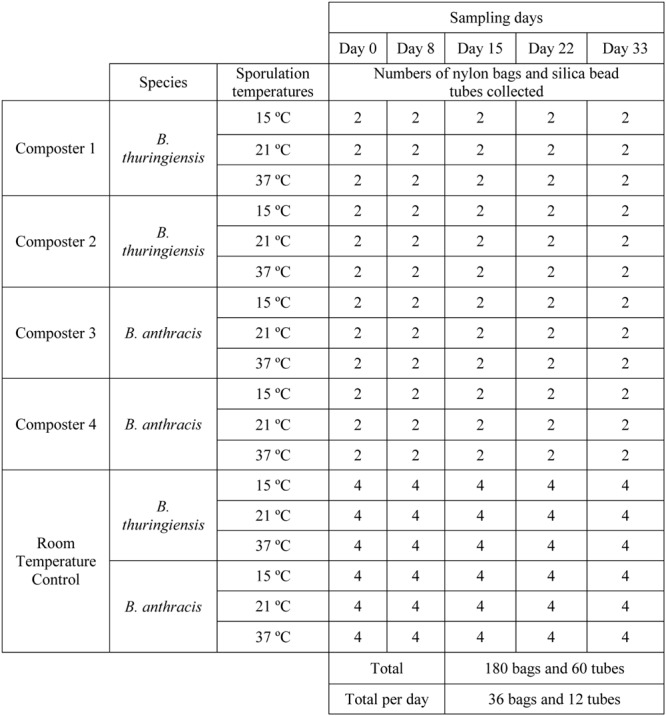
**Schematic representation of the experimental design and numbers of *Bacillus thuringiensis* and *B. anthracis* samples collected during composting under biocontainment conditions.** Duplicated composters were used for each *Bacillus* species sporulated at 15, 21, and 37°C. Manure was inoculated with spore suspensions and then sealed in nylon bags prior to placing into composters. For a room temperature control, autoclaved silica beads were inoculated to achieve the same spore concentration as for inoculation of manure and then sealed in Falcon tubes. Two replicate nylon bags for each sporulation temperature and species were collected from the composters at days 0, 8, 15, 22, and 33. Room temperature controls of duplicate nylon bags and tubes (i.e., total number = 4) for each sporulation temperature and species were collected on the same sampling day.

Passively aerated laboratory-scale composters were used as described by Xu et al. (2010). These 110-L cylindrical polyethylene vessels were sealed and insulated with a 50 mm layer of polyurethane foam. For the purpose of passive aeration, an air plenum (0.1 m height) was created at the bottom of each composter using a perforated polyethylene panel with 10 mm diameter holes. Inlet and outlet air holes (25 mm) were drilled in the side, near the bottom and in the lid to enable passive aeration. Fresh feedlot manure (45 ± 0.1 kg; wet-weight basis) and white spruce (*Picea glauca*) wood shavings (4.5 ± 0.1 kg) were thoroughly mixed in a mortar mixer (12S; Crown construction equipment, Winnipeg, MB, Canada) to form a matrix with a moisture content of 76.0 ± 0.3%. The physicochemical properties of the ingredients are described in **Table 1**. Spores of *B. anthracis* and its surrogate *B. thuringiensis* were composted in a level 3 biocontainment laboratory at the CFIA in Lethbridge, AB (**Figure 1**). Prior to the compost experiment, the lab benches and floors were swab-tested to ensure they were not contaminated with *Bacillus* spores. Identical matrices without inoculation with *Bacillus* spores were composted outside of containment with samples being collected for measurement of physicochemical parameters during the composting process. Experiments inside and outside of containment were started simultaneously with four replicated composters outside containment and two replicated composters for each *Bacillus* species inside containment (**Figure 1**).

**Table 1:** Physicochemical characteristics of materials included in matrices used for laboratory composting under both non-containment and containment conditions.

Parameters^∗^	Cattle manure	Wood shavings
Moisture (%)	81.1 ± 0.5	9.6 ± 0.0
Bulk density (kg m^-3^)	854 ± 6	88 ± 1
Total carbon (%)	41.8 ± 0.9	51.4 ± 0.6
Total nitrogen (%)	2.19 ± 0.07	0.08 ± 0.00
C/N ratio	19.1 ± 0.3	662.2 ± 27.5
pH	7.99 ± 0.16	4.87 ± 0.03
EC (ds m^-1^)	1.16 ± 0.06	0.05 ± 0.00
NH_4_-N (mg kg^-1^)	4237 ± 500	3 ± 1
(NO_2_+NO_3_)-N (mg kg^-1^)	40.1 ± 5.2	6.2 ± 0.0

*^∗^All parameters except moisture and bulk density are expressed on a dry-weight basis (w w^-1^). Moisture and bulk density are expressed on a wet-weight basis. EC, electrical conductivity.*

### Spore Preparation

The strain of *B. anthracis* Sterne was kindly provided by Dr. Elizabeth Golsteyn Thomas (CFIA) and Mr. Doug Bader (Defence Research Development Canada) with NCBI Genome Reference Sequence of NC_005945.1(pXO2-), while *B. thuringiensis* 4A3 was provided by Dr. Tim Lysyk (Agriculture and Agri-Food Canada, Lethbridge Research Centre). Spores of *B. anthracis* Sterne and *B. thuringiensis* 4A3 were prepared from triplicate overnight cultures of single discrete colonies as previously described by Reuter et al. (2011) and Shields et al. (2012), respectively. Briefly, 100 μl of the overnight culture was used to inoculate culture flasks containing 50 ml of tryptic soy agar [Becton Dickinson (BD), Franklin Lakes, NJ, USA) supplemented with 5% sheep blood for *B. anthracis* or AK number 2 agar (BD) supplemented with 20 μg ml^-1^ MgSO_4_ and 80 μg ml^-1^ CaCl_2_ for *B. thuringiensis*. Flasks were incubated at either 15, 21, or 37°C for 8 days until sporulation was complete. Spores were harvested by dispensing phosphate buffered saline (PBS) containing 0.05% Tween 20 (PBST; Sigma–Aldrich, St. Louis, MO, USA) into each flask with gentle mixing in the presence of glass beads to suspend the spores. Suspensions were transferred into 50 ml Falcon tubes and centrifuged at 6,000 × *g* for 10 min at 4°C to pellet the spores. The supernatant was discarded, and the pellets were washed five times with PBST. After the last wash, pellets were resuspended in 50% ethanol and centrifuged as described above to remove all remaining vegetative cells. After washing, a droplet of the suspension was examined using phase-contrast microscopy to ensure that cellular debris and vegetative cells were removed. For each *Bacillus* species, spores generated at each temperature were re-suspended in PBS with 1% bovine serum albumin (BSA; Sigma–Aldrich) and then serially diluted (1:10) for estimating spore CFU as described by Reuter et al. (2011) and Shields et al. (2012).

### Nylon Bag Preparation

Due to the high concentration of *Bacillus* spores in feedlot manure compost matrix, generated spores were inoculated into feces collected directly from the rectum of cattle. The feces had physicochemical properties that were similar to the feedlot manure used in compost, but reduced background microflora for the subsequent detection of *Bacillus* spores (data not shown). Feces (5.0 ± 0.1 g; wet basis) was shaped into spheres and then inoculated with *Bacillus* spore suspensions to achieve ∼7.5 log_10_ CFU g^-1^ manure for each sporulation temperature and *Bacillus* species. However, a lower concentration of *B. thuringiensis* spores (i.e., ∼5.5 log_10_ CFU g^-1^ manure) was used at 37°C due to reduced yield, a response that has been observed for this species when it was sporulated by others at 40°C (Ignatenko et al., 1983). Inoculated manure spheres were sealed in nylon bags (5 × 10 cm; 53 μm pore size; ANKOM Technology, Macedon, NY, USA) prior to placing into composters. For controls, 3 g of autoclaved (121°C, 20 min) silica beads (4 mm; Fisher Scientific, Ottawa, ON, Canada) were inoculated with spore suspensions to achieve the same spore concentration as in manure and then sealed in a sterile 50 ml Falcon tube. Controls of nylon bags containing inoculated manure spheres and tubes containing inoculated silica beads were both retained at room temperature during the experimental period. Duplicate control nylon bags and tubes for each sporulation temperature and *Bacillus* species were collected at each sampling day (**Figure 1**). Nylon bags were prepared on the day of compost construction and implanted into compost immediately after the compost was prepared.

### Nylon Bag Implantation and Sampling Procedures

For each sporulation temperature and *Bacillus* species, two replicate nylon bags were placed in a larger polyester mesh bag (5 mm pore size) along with 200 g of freshly mixed compost. As each composter was filled, four replicate mesh bags for each sporulation temperature were placed at a depth of 30 cm below the surface of the compost matrix, resulting in a total of twelve mesh bags in each composter. Single mesh bags for each sporulation temperature were collected from each composter after 8 and 15 days of composting (**Figure 1**). A total of three mesh bags were removed at each sampling time per replicate composter (**Figure 1**). After collection at day 15, each composter was emptied, and contents were mixed with water to return the compost to its original moisture level. Compost was then returned to its original composter for a second heating cycle. As the composters were refilled, the remaining mesh bags were placed in each composter at the same depth as in the first cycle. In the second composting cycle, mesh bags were collected after 22 and 33 days (**Figure 1**). Compost temperature was continuously measured at the same depth as the mesh bags were implanted (Xu et al., 2010). Composters outside of containment were managed similarly, except that the manure implanted in the compost matrix was not inoculated with *Bacillus* spores. The experiment was designed in this manner as compost within the composters in containment could not be removed from the containment laboratory for chemical analysis.

Compost in each composter outside of containment was collected at day 15 after mixing and moistening, and also from each of the mesh bags for physicochemical analyses. Compost temperatures, oxygen concentration, moisture, bulk density, TC, TN, pH, EC, and mineral N (NH_4_^+^ and NO_2_^-^ + NO_3_^-^) were measured (Xu et al., 2010).

### Enumeration of *Bacillus thuringiensis* and *Bacillus anthracis*

Upon removal of mesh bags from compost, nylon bags were removed from mesh bags and enclosed within a water-tight container. At each sampling day, controls of silica bead tubes and nylon bags retained at room temperatures were also sealed within double packaged Ziplock bags (SC Johnson, Racine, WI, USA). Subsequently, all sealed cups and bags were submerged in bleach (10%) for 30 min and retained within the containment laboratory for enumeration of *Bacillus* spores. Nylon bags of each *Bacillus* species were placed into sterile stomacher bags containing 45 ml PBS and blended for 2 min in a Stomacher 400 (Seward, Davie, Fl, USA) at 230 rpm. A 5 ml aliquot was transferred to a 15 ml Falcon tube and incubated at 75°C for 20 min in a shaking water bath. After cooling, a 100 μl aliquot was serially diluted (10^-1^–10^-5^) into PBS and plated onto duplicate blood tryptic soy agar plates. For silica bead samples, 30 ml of PBS were added into each 50 ml Falcon tube containing the beads and then mixed using a serological pipet. A 5 ml aliquot was then transferred to a 15 ml Falcon tube. The tubes were heat treated, serially diluted and plated as described for nylon bags. Colonies on the plates were enumerated after incubation at 37°C for 16–18 h, and only plates that contained 30 to 300 CFU were counted. The quantification limit was set at ≥30 CFU in the first dilution (10^-1^). Numbers of *B. thuringiensis* and *B. anthracis* spores were calculated as CFU per g of the original weight of manure in the nylon bags or silica beads.

### Statistical Analysis

Numbers of *B. thuringiensis* and *B. anthracis* spores were log transformed before analysis. Changes in the spore number for each *Bacillus* species and temperature profiles during biocontainment composting were analyzed using the MIXED Procedure of SAS (Version 9.2; SAS Institute Inc., Cary, NC, USA) with time treated as a repeated measure in the model. Main effects of sampling day, sporulation temperature and their interaction were considered to be statistically significant at a probability level of <0.05.

## Results

### Compost Properties

**FIGURE 2:**
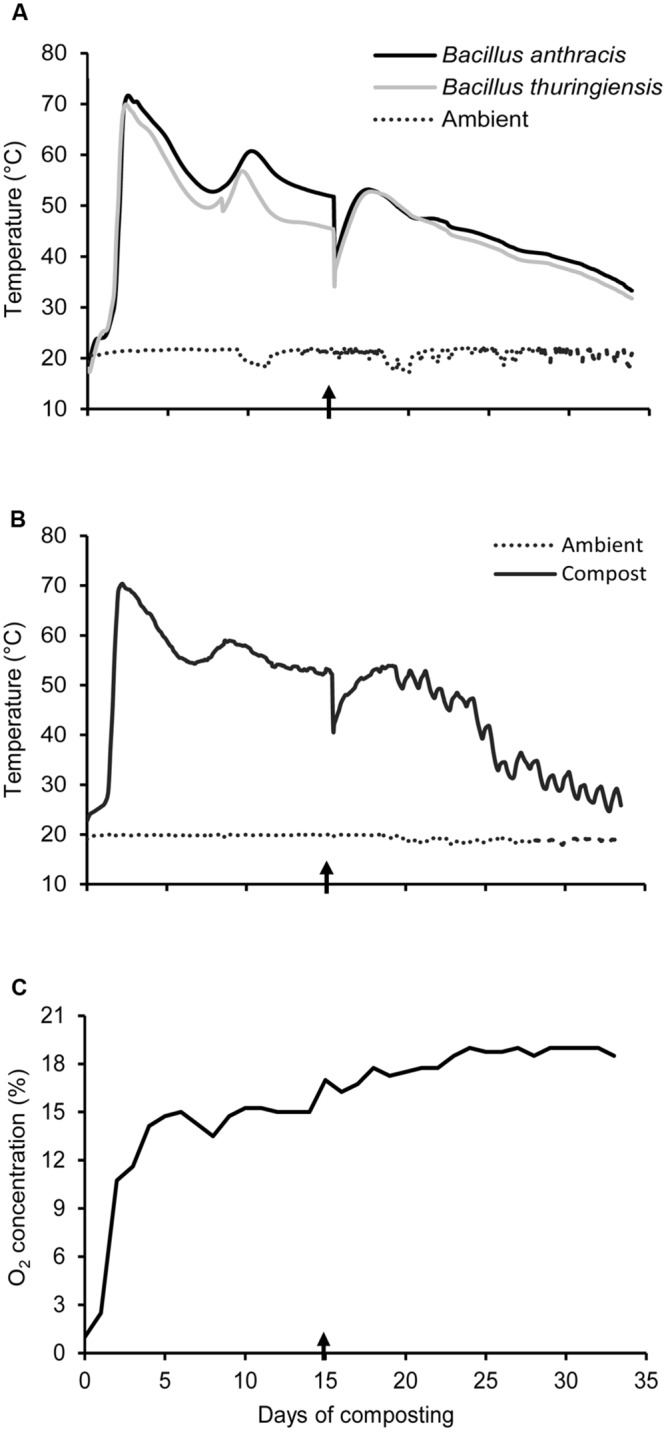
**(A)** Temperature during composting of feedlot cattle manure containing *B. thuringiensis* and *B. anthracis* spores under containment, and **(B)** temperature and **(C)** O_2_ concentration during composting of feedlot cattle manure outside of containment. Arrows indicate the date when compost was mixed and moistened.

Under biocontainment, compost temperatures for each *Bacillus* species were affected (*P* < 0.05) by composting cycle. All the composters heated rapidly, with temperatures peaking after 2 days at 72°C for composters containing *B. anthracis* and 70°C for those containing *B. thuringiensis* (**Figure 2A**). Subsequently, temperatures steadily declined, but increased again and peaked at 61°C for *B. anthracis* at day 10 and 57°C for *B. thuringiensis* at day 9. During the first composting cycle, temperature remained above 55°C for 8 and 5 days for *B. anthracis* and *B. thuringiensis*, respectively (**Figure 2A**). After mixing and moistening of compost on day 15, temperatures did not exceed 55°C and peaked at 53°C on day 17 for both *B. anthracis* and *B. thuringiensis* (**Figure 2A**), which were lower (*P* < 0.05) than the peak temperatures measured in the first cycle.

Compost temperature in containment (**Figure 2A**) mirrored that obtained outside of containment (**Figure 2B**) which was used to assess changes in physicochemical parameters during composting. Oxygen concentration was 1% in compost at day 0 (**Figure 2C**) and then increased to 15% after 5 days, and remained between 15 and 19% until the end of experiment (**Figure 2C**). During the two cycles of composting, moisture content consistently remained in the range of 74–77% (wet weight basis; **Table 2**). Levels of TC, C/N ratio, NH_4_^+^-N, and (NO_2_^-^ + NO_3_^-^)-N steadily declined over each composting cycle, whereas TN tended to increase (**Table 2**). The pH was 7.7 at the start of composting, and increased to 9.1 and 9.3 after the first and second composting cycle, respectively (**Table 2**). Both compost EC and bulk density gradually declined during the initial composting cycle. However, compost EC remained the same whereas bulk density increased over the second cycle (**Table 2**).

**Table 2:** Physicochemical changes of compost mixtures during laboratory-scale composting.

Parameters^∗^	Day 0	Day 8	Day 15 (Before mixing)	Day 15 (After mixing)	Day 22	Day 33
Moisture (%)	75.1 ± 0.6	75.2 ± 1.7	74.3 ± 1.4	77.6 ± 0.5	76.7 ± 1.1	77.0 ± 1.0
Total carbon (%)	46.1 ± 0.3	45.3 ± 0.3	44.8 ± 0.2	44.9 ± 0.5	43.7 ± 0.3	42.6 ± 0.6
Total nitrogen (%)	1.40 ± 0.02	1.57 ± 0.05	1.53 ± 0.03	1.63 ± 0.04	1.82 ± 0.03	2.20 ± 0.05
C/N ratio	33.0 ± 0.5	29.0 ± 1.2	29.4 ± 0.6	27.7 ± 0.9	24.1 ± 0.6	19.4 ± 0.6
pH	7.65 ± 0.06	8.84 ± 0.03	9.11 ± 0.03	8.92 ± 0.02	9.17 ± 0.04	9.25 ± 0.03
EC (ds m^-1^)	1.02 ± 0.05	0.63 ± 0.09	0.63 ± 0.09	0.66 ± 0.07	0.66 ± 0.07	0.66 ± 0.10
NH_4_-N (mg kg^-1^)	2514 ± 15	1229 ± 296	1274 ± 198	1163 ± 64	429 ± 156	192 ± 68
(NO_2_+NO_3_)-N (mg kg^-1^)	33.5 ± 7.8	10.3 ± 3.4	8.4 ± 2.3	12.6 ± 9.5	1.9 ± 0.7	3.4 ± 0.8
Bulk density (kg m^-3^)	491 ± 7	481 ± 17	471 ± 19	552 ± 19	559 ± 19	573 ± 21

*^∗^All parameters except moisture and bulk density are expressed on a dry-weight basis (w w^-1^). Moisture and bulk density are expressed on a wet-weight basis. EC, electrical conductivity.*

### Survival of *Bacillus thuringiensis* Spores

**FIGURE 3:**
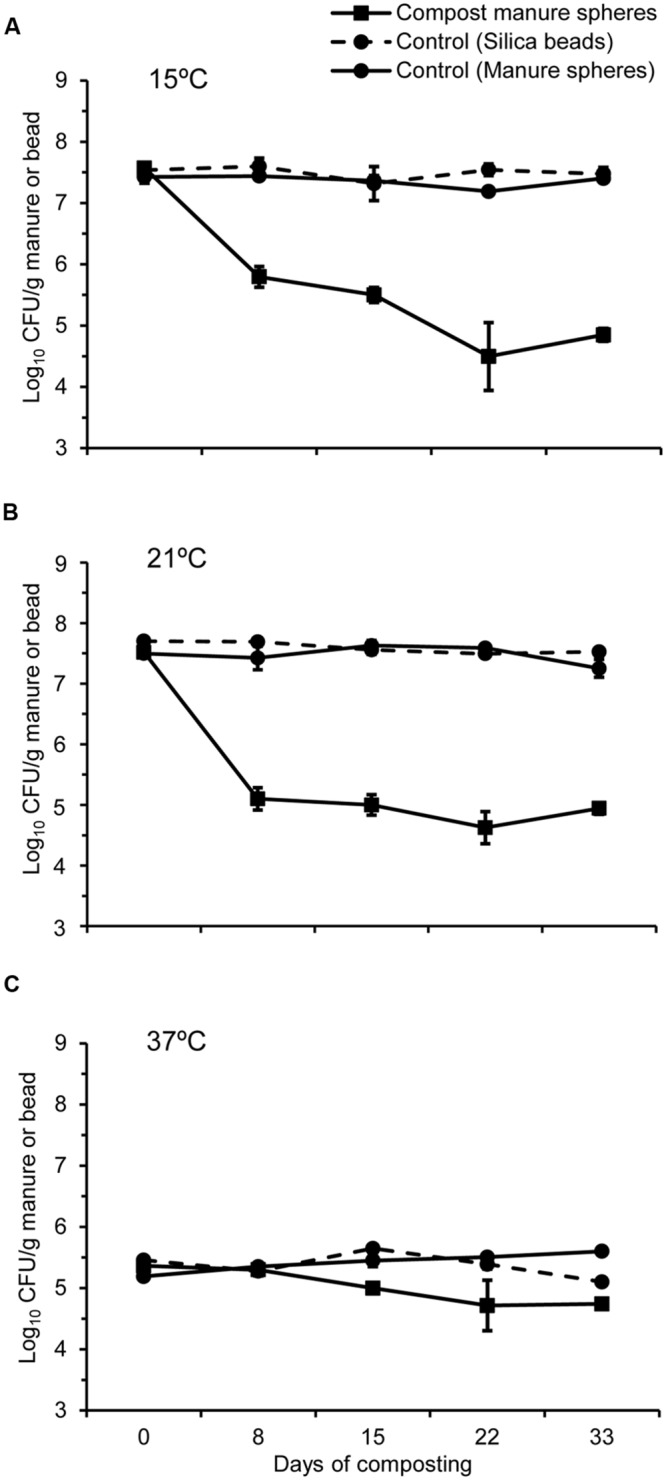
**Survival of *B. thuringiensis* spores (Log_10_ CFU g^-1^ manure) generated at different temperatures **(A)** 15°C, **(B)** 21°C, and **(C)** 37°C and placed in manure spheres that were composted with feedlot cattle manure for 33 days.** For controls, silica beads and fresh manure spheres were inoculated with *B. thuringiensis* spores and kept at room temperature over 33 days.

For controls, *B. thuringiensis* spores generated at all three temperatures remained relatively stable within the silica beads and manure at room temperature, only declining by 0–0.3 log_10_ CFU g^-1^ over 33 days (**Figure 3**). During two cycles of composting, viability of *B. thuringiensis* spores sporulated at 15 and 21°C exhibited a similar reduction of 2.7 and 2.6 log_10_ CFU g^-1^, respectively (**Figures 3A,B**). This reduction was more (*P* < 0.05) pronounced in the first than the second composting cycle (**Figures 3A,B**). Overall, the reduction in viability of spores generated at 37°C averaged 0.6 log_10_ CFU g^-1^ after two cycles of composting (**Figure 3C**), which was lower than (*P* < 0.05) those sporulated at either 15 or 21°C (**Figures 3A,B**).

### Survival of *Bacillus anthracis* Spores

**FIGURE 4:**
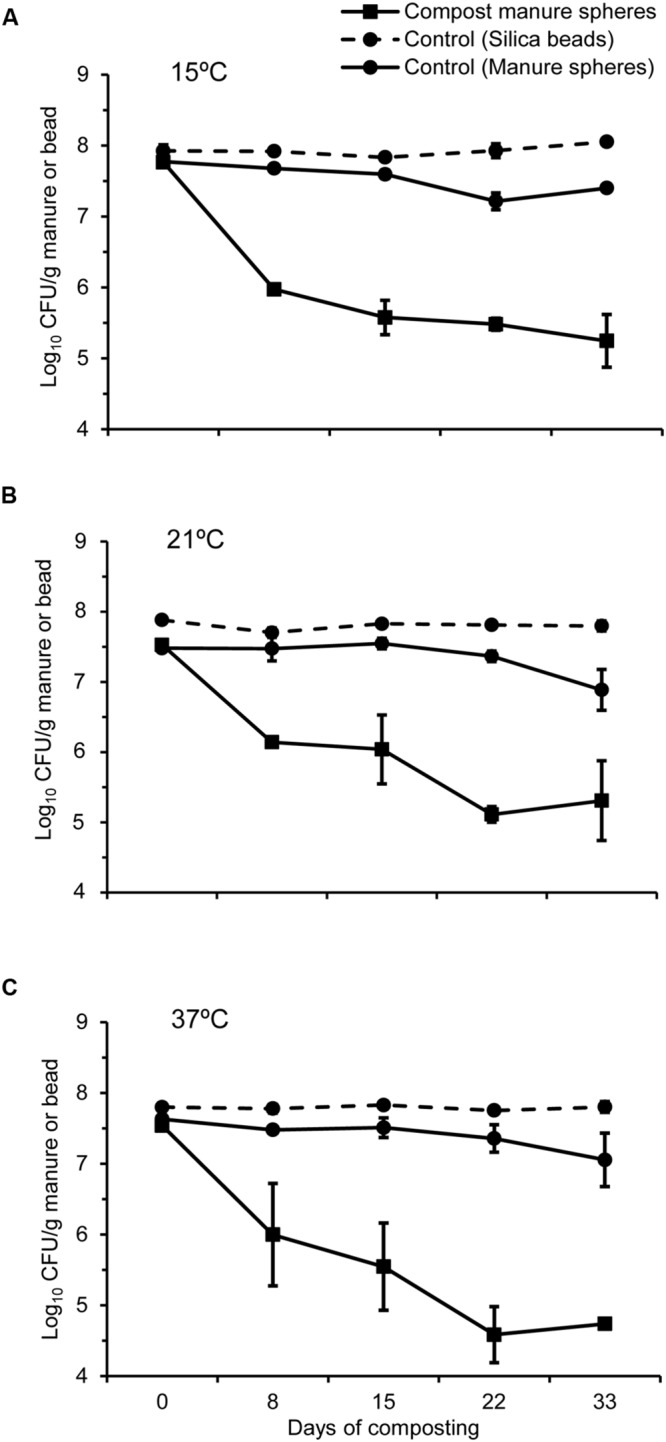
**Survival of *B. anthracis* spores (Log_10_ CFU g^-1^ manure) generated at different temperatures **(A)** 15°C, **(B)** 21°C, and **(C)** 37°C and placed in manure spheres that were composted with feedlot cattle manure for 33 days.** For controls, silica beads and fresh manure spheres were inoculated with *B. anthracis* spores and kept at room temperature over 33 days.

Spores of *B. anthracis* generated at all three sporulation temperatures remained virtually stable in the control silica beads stored at room temperature over 33 days (**Figure 4**). In contrast, spore viability decreased by 0.4–0.6 log_10_ CFU g^-1^ in the control manure over the same time period (**Figure 4**). Composting reduced the number of viable spores compared to inoculated control samples held at room temperature. However, sporulation temperature exerted no effect on the survival of *B. anthracis* spores in compost (**Figure 4**). The numbers of viable spores generated at 15, 21, and 37°C declined by 2.2, 1.5, and 2.0 log_10_ CFU g^-1^, respectively, after the first composting cycle (**Figures 4A–C**). After the compost was mixed and moistened, numbers of viable *B. anthracis* spores generated at all three temperatures continued to decline, with reductions of 2.5, 2.2, and 2.8 log_10_ CFU g^-1^ at 15, 21, and 37°C, respectively (**Figures 4A–C**).

## Discussion

Passively aerated laboratory-scale composters (Xu et al., 2010) were used to study the composting of *B. anthracis* under containment conditions. In this study, physicochemical changes in compost over the experimental period were similar to those that we have reported in previous studies (Xu et al., 2010, 2013, 2014), demonstrating that our laboratory-scale composters were appropriate as a model for examining the ability of composting to inactivate *B. anthracis* spores. Using the same model, our laboratory has investigated the degree of degradation of scrapie (Xu et al., 2013, 2014), CWD and BSE (Xu et al., 2014), showing a 1–3 log_10_ reduction in these prions after two cycles of composting. In this study, our findings further demonstrate a 2–3 log_10_ reduction of *B. anthracis* spores after two cycles of composting. Due to biocontainment restrictions for field-scale composting of *B. anthracis*, it was necessary to first assess the inactivation of *B. anthracis* spores within biocontained laboratory composting systems.

Several techniques, alone or in combination, have been tested for the inactivation of *Bacillus* spores, including heating, radiation, UV treatment, chemicals, and high pressure (Roberts and Hoover, 1996; Setlow, 2006; Xing et al., 2014). The mechanisms involved in these inactivation methods are linked to physicochemical modifications of metabolism proteins, inactivation of critical enzymes essential for spore germination, accumulative DNA damage, breakdown of spore membrane permeability barriers, and flattening and lengthening of the spores (Cléry-Barraud et al., 2004; Coleman et al., 2010). Composting is a natural biological process involving decomposition and stabilization of organic matter within an aerobic environment. Achieving optimal temperatures in compost is critical to reducing the viability of *Bacillus* spores. The majority of pathogens in compost are rendered non-viable if exposed to temperatures above 55°C for an extended period of time (Kalbasi et al., 2006; Xu et al., 2009). Guidelines for composting from the Canadian Council of Ministers of the Environment (CCME, 2005) and the United States Environmental Protection Agency (USEPA, 1995) both suggest that the duration of exposure at or above 55°C should be at least 15 consecutive days within compost windrows and 3 consecutive days in confined or in-vessel composters. Our recent findings showed that sustained thermophilic temperatures for 78 of 150 days in a laboratory oven reduced the number of viable *B. licheniformis and B. thurigiensis* spores in cattle manure by ≥5 log_10_ CFU g^-1^ (Stanford et al., 2016). In the present study, temperature profiles showed that compost temperature remained ≥55°C for an average of 7 days, resulting in 2–3 log_10_ CFU g^-1^ reduction of *B. anthracis* spores in compost. Although temperatures did not exceed 55°C in the second heating cycle, mixing and moistening of compost extended the period for inactivation of *B. anthracis* spores. However, the reduction of *B. anthracis* spores was lower in the second cycle as compared to the first cycle. Peak temperature was higher and the duration of exposure to thermophilic temperatures was longer in the first as compared to the second composting cycle, suggesting that thermophilic compost temperatures ≥55°C were more effective at reducing spore viability than mesophilic temperatures. This is consistent with our previous results on the inactivation of *B. cereus* as a surrogate for *B. anthracis* during composting of cattle carcasses for 217 days (Stanford et al., 2015).

Moreover, the duration of survival of *Bacillus* spores is lower when exposed to wet heat compared to dry heat, although the mechanisms for this phenomenon have not been fully elucidated (Nicholson et al., 2000). Our laboratory-scale compost remained moist (i.e., ∼76% moisture content; wet weight basis) as we added water to the compost after the first cycle. This approach likely increased the inactivation of *Bacillus* spores as compared to the scenario where dehydration would lead to a reduction in composting activity. Field scale compost piles consist of a heterogeneous matrix of organic matter with significant variation in moisture content within the mass, particularly with static composting (Xu et al., 2009). The decline in spore viability may be less in regions of the pile where moisture levels are suboptimal for composting. In addition, pH has been reported to influence the heat resistance of *Bacillus* spores. Heat resistance is greatest at near neutrality pH and decreases under acid or alkaline conditions (Palop et al., 1999). Baweja et al. (2008) showed that the viability of *B. anthracis* Sterne spores was reduced by exposure to either acidic or alkaline chemicals. In this study, compost pH increased by more than 1.5 units to a pH ∼9, conditions that likely enhanced the inactivation of *B. anthracis* spores in compost.

*Bacillus anthracis* belongs to the *B. cereus* group along with *B. cereus, B. mycoides, B. pseudomycoides, B. thuringiensis*, and *B. weihenstephanensis* with which it shares many morphological, biochemical, and genetic similarities (Harrell et al., 1995; Maughan and Van der Auwera, 2011). Our laboratory has previously used *B. cereus, B. licheniformis*, and *B. thuringiensis* as surrogates for *B. anthracis* to assess the survival of *B. anthracis* spores during long term composting (i.e., ∼200 days) of cattle carcasses (Reuter et al., 2011; Stanford et al., 2015). Recent studies have shown that *B. thuringiensis* is gaining acceptance as the most suitable model for *B. anthracis* (Greenberg et al., 2010; Bishop and Robinson, 2014; Tufts et al., 2014). One of the genetic differences between these two species is that *B. anthracis* lacks a gene encoding a pleiotropic regulator, which is involved in sensing the external environment, activation of exoenzyme synthesis and other functions related to spore survival within the environment (Gohar et al., 2008; Bishop, 2014). However, this difference does not appear to result in differences between these two species in their ability to cope with environmental stresses, including dry heat, wet heat, and chlorination (Rice et al., 2005; Buhr et al., 2012; Setlow et al., 2014). Our results demonstrated that *B. thuringiensis* spores were inactivated in a manner similar to *B. anthracis* by composting, further supporting the use of *B. thuringiensis* spores as a model to assess the environmental robustness of *B. anthracis* spores.

To date, little is known about the effect of the sporulation temperature on survival of *Bacillus* spores during the composting process. Studies with various *Bacillus* species have shown that increasing the temperature during sporulation correlates with increased heat resistance of *B. cereus* (Collado et al., 2006), *B. weihenstephanensis* (Baril et al., 2011), *B. licheniformis* (Raso et al., 1995), and *B. subtilis* (Condon et al., 1992). However, this trend can be inconsistent among different strains within the same species (Fernandez-Coll and Rodriguez-Toro, 1986; González et al., 1999), indicating that there are likely genotypic differences among strains that impact the heat resistance of *Bacillus* spores (Condon et al., 1992; Raso et al., 1995). Results in our study are consistent with Ignatenko et al. (1983) who reported that a rise in sporulation temperature from 20 to 35°C increased the thermal resistance of *B. thuringiensis* spores. For species of *B. anthracis*, Baweja et al. (2008) demonstrated that *B. anthracis* Sterne spores generated at 45°C were more resistant to wet heat than those sporulated at 25°C. However, *B. anthracis* spores sporulated at temperatures as high as 37°C in the present study did not exhibit superior survival during composting as compared to those that were formed at lower temperatures. The complex environmental conditions during composting likely make temperature exposure only one of many factors that influence the viability of *B. anthracis* spores and it is possible that strains of *B. anthracis* differ in responses to these environmental challenges. Investigating the composting of endospores exposed to different sporulation temperatures is crucial, as it has important implications concerning decontamination protocols under various weather conditions (Condon et al., 1992; Palop et al., 1999). Our findings suggest that composting may be suitable for disposal of *B. anthracis* infected carcasses at geographical locations experiencing mild (15–21°C) or hot (>30°C) ambient temperatures. There is evidence that *B. anthracis* spores may undergo germination and growth in plant rhizosphere (Saile and Koehler, 2006) and may grow and amplify within amoebic hosts that remain viable outside of this temperature range (Dey et al., 2012).

To our knowledge, this is the first study describing the inactivation of *B. anthracis* spores as a result of composting. Limited biomass in the laboratory-scale composters resulted in thermophilic temperatures (≥55°C) for only 7 days during two composting cycles over a period of 33 days. This contrasts with our previous field-scale system employed for investigation of the same strain of *B. thuringiensis* spores where temperatures ≥55°C were recorded for 75 days out of 230 days of composting (Reuter et al., 2011). These differences in duration of thermophilic temperatures were reflected in the degree of inactivation of *B. thuringiensis* spores with a 1–3 log_10_ reduction in the laboratory composters after 33 days, as compared to a ∼5 log_10_ reduction within field-scale composters over 112 days (Reuter et al., 2011). Therefore, it is not unrealistic to expect a more extensive inactivation of *B. anthracis* spores during field-scale composting. However, the complete inactivation of *B. anthracis* spores during composting is likely unrealistic. In practice, compost piles are affected by a number of internal and external factors including the heterogeneous nature of animal tissues and other matrix components that can result in fluctuating heat generation and distribution (Xu et al., 2009). Microbial communities that may play a role in the inactivation of spores may also differ among locations within the compost pile (Tkachuk et al., 2014). Consequently, further research is required to investigate the survival of *B. anthracis* spores in the microenvironments of compost piles where thermophilic temperature conditions may be compromised.

In this study, *B. anthracis* Sterne, an attenuated non-encapsulated variant, was employed (Welkos and Friedlander, 1988). This strain does not have the pXO2 plasmid, which carries the capsule genes. However, all chromosomal genes responsible for sporulation and germination are present (Cléry-Barraud et al., 2004). Thus, spore components and spore resistance of this strain are assumed to be identical to those of the wild strain (Pézard et al., 1993). However, further comparisons of the difference in the survival of spores from the wildtype *B. anthracis* strain and Sterne strain may be required to assess the potential use of composting for disposal of *B. anthracis* infected carcasses. Furthermore, heat resistance of *Bacillus* spores is affected by the nature of matrix in which spores are heated (Coroller et al., 2001). Our findings showed that the numbers of viable *B. anthracis* spores in manure retained at room temperatures declined by ∼0.5 log_10_ over 33 days while spores in the silica beads remained stable over the same time period. This suggests that the majority of the reduction in spore viability was associated with exposure to high temperatures, but microbial activity may have also contributed to this response. It is possible that the thermal resistance of *B. anthracis* spores originating from carcass exudate may differ from those composted in manure (Stanford et al., 2015). Future studies should evaluate the survival of *B. anthracis* spores in compost in the presence of animal tissues or fluids with various levels of fat, carbohydrate or proteins. However, we have previously shown that tissues other than ossified bones in bovine carcasses are completely degraded during field scale composting (Xu et al., 2009).

## Conclusion

The composting should be considered as a simple method for on-site containment of infected carcasses in the event of an anthrax outbreak. The outcomes from this study showed a 3 log inactivation of *B. anthracis* spores was achieved after 1 month of laboratory-scale composting. Further reductions in survival of *B. anthracis* spores are likely possible with field-scale composting as the duration of the thermophilic period is typically much longer. Although all *B. anthracis* spores might not be completely destroyed by composting, potential for the spread of these spores at infectious doses after composting would be reduced after land application due to both dilution and inactivation effects. Therefore, carcass composting might be considered as a viable method to reduce the dissemination of *Bacillus* spores to the surrounding environment.

## Author Contributions

SX: Designed/conducted laboratory experiments/first draft manuscript. AH: Designed/conducted laboratory experiment. RB: Conducted laboratory experiments. TR: Data analysis/contributed to manuscript. KA: Experimental design/laboratory facilities support for Level 3/funding of research activities. LS: Contributed to manuscript/experimental design. TM: Principal investigator for project/experimental design/finalized draft of manuscript/provided funding.

## Conflict of Interest Statement

The authors declare that the research was conducted in the absence of any commercial or financial relationships that could be construed as a potential conflict of interest.

## Abbreviations

TC: total carbon
TN: total nitrogen
EC: electrical conductivity
